# Pediatric palliative care in practice: real-world service utilization and care pathways in a regional Italian health system

**DOI:** 10.1007/s00431-026-07386-4

**Published:** 2026-09-08

**Authors:** Marco Gabrielli, Giulia Zamagni, Sara Persello, Valeria Tozzi, Francesca Peri, Lucia De Zen

**Affiliations:** 1 Department of Medicine, Surgery and Health Sciences, University of Trieste, Trieste, Italy; 2 Division of Government, Health and Non-Profit, Bocconi University, Milan, Italy; 3 Institute for Maternal and Child Health, “IRCCS Burlo Garofolo”, Trieste, Italy

**Keywords:** Pediatric palliative care, Healthcare utilization, Technological dependency, Integrated care pathways

## Abstract

The purpose of this study was to describe real-world pediatric palliative care (PPC) utilization and care pathways within a regional Italian health system and to compare the observed specialist PPC caseload with the expected regional need. This retrospective observational study included pediatric patients formally taken in charge by the tertiary-level PPC Hub Center at IRCCS Burlo Garofolo, Trieste, between January 2022 and December 2025. Electronic clinical records were reviewed to reconstruct demographic and clinical characteristics, technological dependency, healthcare contacts, home-based activities, territorial mobility, and expected regional PPC need. Among 159 screened patients, 91 met the inclusion criteria. Most patients were non-oncologic (*n* = 80) and showed high clinical complexity and frequent technological dependency, with 29.7% requiring more than three medical devices. Median age was 6 years (IQR 1–12), foreign citizens accounted for 26.4% of the cohort, and overall mortality was 29.7%. Median healthcare utilization was 61 accesses per patient (IQR 35–97), with broad multidisciplinary involvement across hospital, outpatient, emergency, and home-based services. Care delivery was strongly centralized toward the tertiary PPC Hub Center. Observed specialist PPC coverage was 42.3% of the estimated regional specialist need, with lower coverage in more distant Local Health Authorities.

*Conclusion*: Children receiving specialist PPC showed substantial clinical complexity, technological dependency, and intensive multidisciplinary healthcare use. Hub-centered pathways and the coverage gap support strengthening territorial capacity, proactive case identification, culturally sensitive family support, and integrated Hub-and-Spoke governance.

**Table:** 

**What is Known:** • *Children needing PPC have complex, long-term needs and high healthcare utilization.* • *Access to specialist PPC remains uneven across Italian regions and care settings.*	
**What is New:** • *This regional real-world study reconstructs longitudinal PPC pathways across hospital, home, and territorial services.* • *Specialist PPC coverage reached 42.3% of estimated need, with marked LHA variability and hub-centered care.*

**Supplementary Information:**

The online version contains supplementary material available at https://doi.org/10.1007/s00431-026-07386-4.

## Introduction

Advances in pediatric medicine and life-sustaining technologies have improved survival among children with complex, incurable, and severely disabling conditions [1]. Although children with medical complexity (CMC) represent a small proportion of the pediatric population, they account for a disproportionate share of healthcare utilization, hospital days, costs, and in-hospital mortality [2, 3]. Within this population, children eligible for pediatric palliative care (PPC) form a subgroup with particularly complex and multidimensional needs, often requiring recurrent hospital contacts, emergency care, specialist consultations, technological support, and continuity between hospital and community services [4, 5]. PPC aims to improve quality of life through early, integrated, and multidisciplinary care that extends beyond end-of-life support and accompanies patients and families throughout the disease trajectory [1, 6]. Access to specialist PPC has been associated with reduced hospital utilization, fewer intensive care unit days, lower healthcare costs, and a lower likelihood of in-hospital death, particularly in the last year of life [7]. Nevertheless, PPC availability and organization remain uneven across Italy, reflecting the regionalized structure of the National Health Service and persistent gaps in territorial coverage, staffing, continuity of care, and 24/7 availability [1, 8]. Most evidence on pediatric complexity and PPC still focuses on single care settings, such as hospital admissions, emergency department visits, or individual referral centers, rather than on longitudinal pathways across hospital, community, specialist, and private services [9–12]. Clinical tools such as the ACCAPED scale and the Paediatric Palliative Screening Scale support the assessment of eligibility and needs [13, 14]; the ACCAPED scale is a validated multidimensional tool assessing ten domains of clinical and care complexity (respiration, feeding, seizures or altered consciousness, skin integrity, mobility, communication, rest and sleep, continence and evacuation, drug administration, and pain), together with a modified “surprise question.” Its total score defines three levels of care: level 1(basic care; ≤ 29 points), level 2 (general PPC; 30–49 points), and level 3 PPC (specialized; ≥ 50 points). The PaPaS is a screening tool assessing five domains (disease trajectory, treatment burden, symptom burden, patient and family needs and preferences, and estimated life expectancy) and similarly identifies increasing PPC involvement: level 1 (introduce PPC; 10–14 points), level 2 (initiation PPC; 15–24 points), and level 3 (PPC as focus of care; ≥ 25 points). Both tools are available in Italian-language versions. However, real-world evidence for clinical governance and system-level planning remains limited. Friuli-Venezia Giulia provides a relevant regional case. Since 2019, pediatric services have been organized through a Hub-and-Spoke PPC network, with a specialist Hub at IRCCS Burlo Garofolo in Trieste and three LHA functioning as spoke centers [15]. This study describes real-world healthcare utilization among children followed by the regional PPC Hub, reconstructs longitudinal care pathways, and compares the observed specialist PPC caseload with expected regional need.

## Methods

### Study design and setting

A retrospective observational study was conducted at the tertiary-level PPC referral center of IRCCS Burlo Garofolo, Trieste, over a 4-year period from January 2022 to December 2025. This timeframe was selected to capture a phase of relative stability in regional PPC delivery, after the establishment of the specialist service in 2019 and the organizational disruptions related to the COVID-19 pandemic. The study was conducted within the Friuli-Venezia Giulia regional PPC network. In this Hub-and-Spoke model, IRCCS Burlo Garofolo acts as the regional PPC Hub, providing specialist care for ACCAPED level 3 patients, multidisciplinary care planning, training, research, and network coordination. The specialist PPC team currently includes three pediatricians, two nurses, one physiotherapist, one speech and language therapist, one psychologist, and one spiritual care provider. Nurses play a central role in symptom monitoring, caregiver education, management of technological dependencies, coordination of home-based care, and early identification of clinical deterioration, in close collaboration with hospital and territorial services. The three Local Health Authorities (ASUFC, ASFO, and ASUGI) function as territorial spokes, ensuring ACCAPED level 1–2 PPC, general pediatric care, and home-care services. This shared-care model combines specialist expertise with continuity of care close to the child’s place of residence.

### Eligibility criteria and study population

Eligibility was assessed using electronic clinical records from the PPC Hub Center. Patients were included if they were formally and continuously taken in charge within the specialist PPC pathway and had at least one documented clinical evaluation between 2022 and 2025. Formal take-charge corresponded to level 3 PPC according to the ACCAPED scale Patients were excluded if they lived outside the region or if their contacts were limited to psychological consultation, pain therapy, or home nursing care only, as these activities were considered supportive components rather than comprehensive PPC management. The upper age threshold was set at 20 years to reflect real-world clinical practice, since some young adults with childhood-onset complex conditions remain under pediatric follow-up during transition to adult services [16, 17].

### Data sources and variables

Data were retrospectively extracted from electronic clinical records held by the PPC Hub Center. For each included patient, investigators reconstructed longitudinal care trajectories across the 4-year observation period. Healthcare utilization was described using the annual volume of recorded contacts, aggregated by patient, year, type of service, and care setting. Collected variables included demographic and clinical characteristics, vital status, oncologic or non-oncologic condition, LHA, category of life-limiting or life-threatening condition, medical device use, private service use, home-based PPC activities, emergency department visits, pediatric inpatient and outpatient contacts, specialist services and initial and final care setting. ACCAPED level 3 was considered the defining criterion for high clinical complexity. Conditions were classified according to the Association for Children with Life-Threatening or Terminal Conditions and their Families framework [18]. To provide a more detailed clinical characterization, non-oncologic conditions were further grouped according to the presence of one or more of the following: neurological disability, acquired conditions, neuromuscular disease, and known genetic diagnoses; oncologic conditions were classified as hematologic malignancies, central nervous system (CNS) tumors, or non-CNS solid tumors. Duration of specialist PPC follow-up was calculated from the date of formal take-charge to the end of follow-up or death. Expected regional PPC need was estimated using published prevalence figures applied to ISTAT regional population data, stratified by LHA [19, 20].

### Ethical considerations

Given the retrospective design and the use of pre-existing data collected during routine care, formal Ethics Committee approval was not required. Data processing complied with the Italian Data Protection Authority Authorization No. 9/2014 and subsequent applicable regulations. All data were analyzed in anonymized and aggregated form to ensure patient confidentiality [21].

## Results

### Study population

Of 159 screened patients, 91(57%) met the inclusion criteria and were retained in the analytical cohort. The median age was 6 years (IQR 1–12), with 31 patients aged 0–12 months (34.0%) and 6 aged 13 months–2 years (6.6%). Overall, 80 patients were non-oncologic (88%) and 11 were oncologic (12%). Foreign citizens accounted for 26.4% of the cohort, and 27 patients died during follow-up, corresponding to an overall mortality of 29.7% (Table 1).

**Table 1:** Demographic and clinical characteristics of the study population

Variables	Total (*n* = 91)	Non-oncologic (*n* = 80)	Oncologic (*n* = 11)
Age, median (IQR)	6 (1–12)	5 (1–11)	11 (1–13)
Age group, n (%)			
0–12 months	31 (34.0%)	28 (35.0%)	3 (27.3%)
13 months–2 years	6 (6.6%)	6 (7.5%)	0 (0.0%)
3–5 years	7 (7.7%)	6 (7.5%)	1 (9.1%)
6–11 years	23 (25.3%)	20 (25.0%)	3 (27.3%)
12–17 years	20 (22.0%)	17 (21.2%)	3 (27.3%)
≥ 18 years	4 (4.4%)	3 (3.7%)	1 (9.1%)
Foreign citizen, n (%)	24 (26.4%)	23 (28.7%)	1 (9.1%)
Complexity (ACCAPED scale), n (%)			
Level 3	91 (100.0%)	80 (100.0%)	11 (100.0%)
Category according to ATS classification, n (%)			
1	16 (17.6%)	5 (6.2%)	11 (100.0%)
2	9 (9.9%)	9 (11.2%)	0 (0.0%)
3	44 (48.4%)	44 (55.0%)	0 (0.0%)
4	22 (24.2%)	22 (27.5%)	0 (0.0%)
Devices, n (%)			
None	12 (13.2%)	8 (10.0%)	4 (36.4%)
One	20 (22.0%)	15 (18.7%)	5 (45.5%)
Two	18 (19.8%)	17 (21.2%)	1 (9.1%)
Three	14 (15.4%)	14 (17.3%)	0 (0.0%)
More than three	27 (29.7%)	26 (32.5%)	1 (9.1%)
Deceased, n (%)	27 (29.7%)	17 (21.2%)	10 (90.9%)
LHA, n (%)			
ASUGI	39 (42.9%)	36 (45.0%)	3 (27.3%)
ASUFC	37 (40.7%)	32 (40.0%)	5 (45.5%)
ASFO	15 (16.5%)	12 (15.0%)	3 (27.3%)
Private service, n (%)	20 (22.0%)	20 (25.0%)	0 (0.0%)
Total accesses*, median (IQR)	61 (35–97)	60 (34–93)	68 (49–147)
Total annualized accesses, median (IQR)	13 (9–22)	12 (9–22)	16 (3–17)

*Abbreviations*: *IQR* interquartile range, *LHA* Local Health Authority

Annotations: *: calculated on the overall time period

Clinical complexity was high. Most patients belonged to category 3 or category 4 conditions accounting for 48.4% and 24.2% of the cohort, respectively. Among non-oncologic patients, category 3 was the most frequent condition group (*n* = 44; 55.0%). All included patients were classified as requiring specialist PPC, corresponding to ACCAPED level 3.

### Technological dependency and service use

**Fig. 1:**
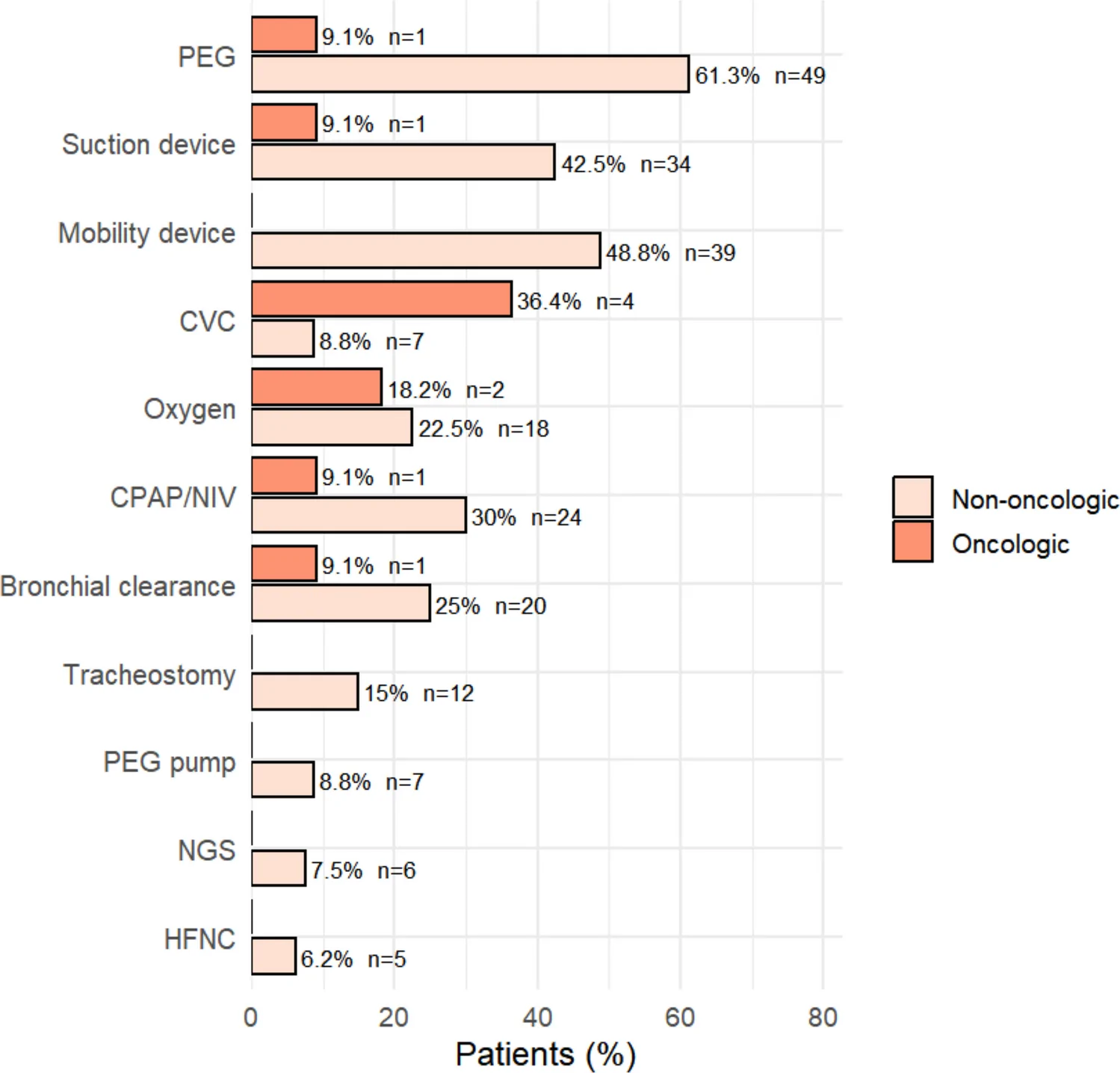
Device utilization among non-oncologic and oncologic patients (PEG: Percutaneous Endoscopic Gastrostomy. CVC: Central Venous Catheter. CPAP: Continuous Positive Airway Pressure NIV: Non-Invasive Ventilation. NGS: Nasogastric Tube. HFNC = High-Flow Nasal Cannula)

Technological dependency was frequent. Only 12 patients (13.2%) had no medical devices, while 27 (29.7%) required more than three devices. Device profiles differed between non-oncologic and oncologic patients. Non-oncologic patients showed a broader and more complex pattern of technological dependency, mainly involving nutritional, respiratory, mobility, and secretion-management supports. In contrast, oncologic patients had a more limited device profile, predominantly related to vascular access and oxygen support (Fig. 1).

Across the study period, patients had a median of 61 healthcare accesses across the regional healthcare system (IQR 35–97), corresponding to approximately 15.25 accesses per patient-year. Service utilization was predominantly concentrated in the medical area in both groups. Among non-oncologic patients, the medical area accounted for 2799 contacts, followed by the surgical area (*n* = 1258) and the emergency area (*n* = 686). The most frequently accessed services were the Emergency Department (*n* = 599), Otolaryngology (*n* = 550), Pediatrics Outpatient (*n* = 514), Pediatrics Inpatient (*n* = 443), Neuropsychiatry (*n* = 428), General Surgery (*n* = 419), and Cardiology (*n* = 349). Among oncologic patients, the medical area accounted for 785 contacts, followed by the surgical area (*n* = 177) and the emergency area (*n* = 78). The most frequently accessed services were hemato-oncology (*n* = 268), pediatrics outpatient (*n* = 232), anesthesia (*n* = 85), emergency department (*n* = 75), cardiology (*n* = 65), otolaryngology (*n* = 60), and ophthalmology (*n* = 60). Further details are provided in the interactive Sankey diagram available as supplementary material.

### Territorial mobility and home-based activities

**Fig. 2:**
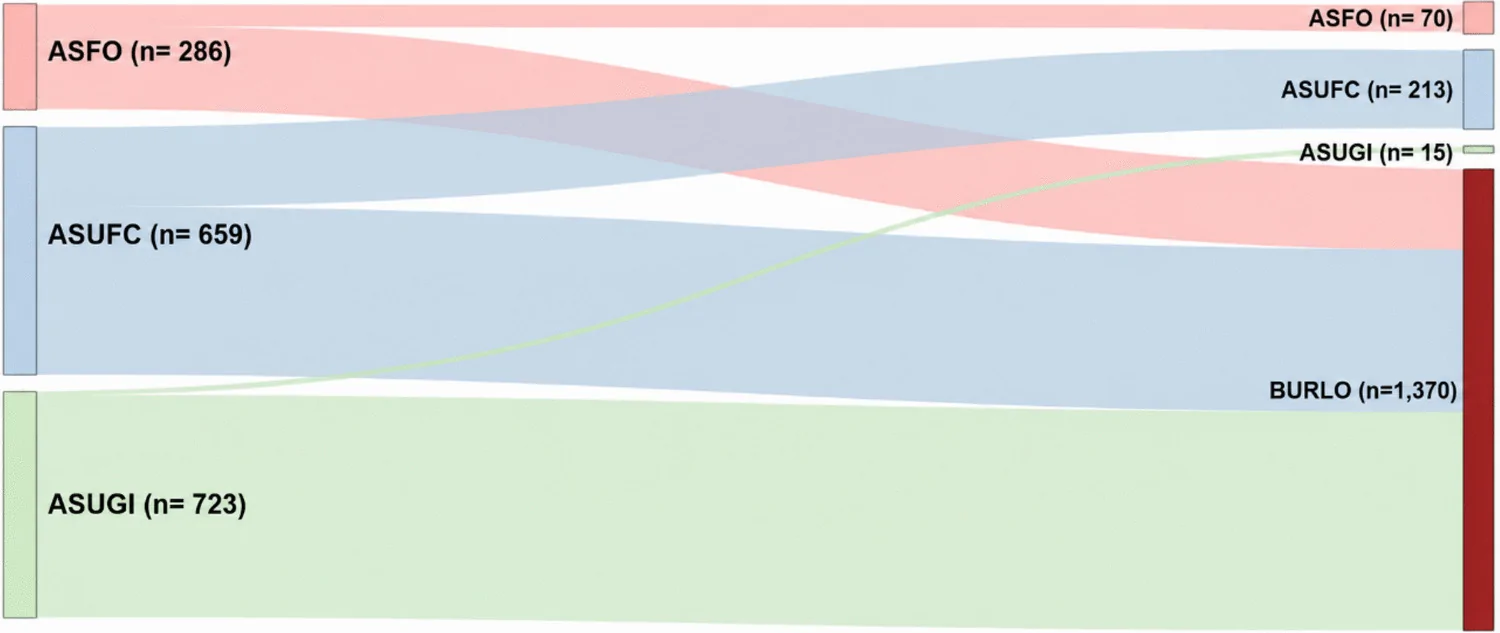
Territorial mobility across Local Health Authorities and final care setting. Each flow represents the number of healthcare contacts originating from the patient’s LHA of residence and directed to the corresponding final care setting during the study period (January 2022–December 2025); flow width is proportional to the number of contacts (ASFO, Azienda Sanitaria Friuli Occidentale; ASUFC, Azienda Sanitaria Universitaria Friuli Centrale; ASUGI, Azienda Sanitaria Universitaria Giuliano Isontina; BURLO: IRCCS Istituto di Ricovero e Cura a Carattere Scientifico)

Patients were distributed across the three LHA: 39 in ASUGI (42.9%), 37 in ASUFC (40.7%), and 15 in ASFO (16.5%). Care pathway analysis showed a clearly hub-centered model of final care delivery, with most contacts converging toward IRCCS Burlo Garofolo irrespective of the initial LHA. Territorial spoke centers accounted for a comparatively smaller share of final contacts, suggesting substantial reliance on the specialist Hub for care coordination and delivery across the regional network (Fig. 2). Home-based and outpatient PPC activities differed between groups (Table 2).

**Table 2:** Distribution of PPC activities among non-oncologic and oncologic patients by year

	Non-oncologic patients				
Service	Total *n* = 821	2022 *n* = 52	2023 *n* = 156	2024 *n* = 194	2025 *n* = 419
Home visit	220 (26.8%)	24 (46.2%)	73 (46.8%)	58 (29.9%)	65 (15.5%)
Home blood sampling	41 (5.0%)	7 (13.5%)	21 (13.5%)	7 (3.6%)	6 (1.4%)
Telemedicine	161 (19.6%)	6 (11.5%)	15 (9.6%)	56 (28.9%)	84 (20.0%)
PPC outpatient clinic	295 (36.0%)	15 (28.8%)	39 (25.0%)	58 (29.9%)	183 (43.7%)
Psychological counseling	104 (12.7%)	0 (0.0%)	8 (5.1%)	15 (7.7%)	81 (19.3%)
	Oncologic patients				
Service	Total *n* = 80	2022 *n* = 2	2023 *n* = 5	2024 *n* = 36	2025 *n* = 37
Home visit	47 (58.8%)	1 (50.0%)	3 (60.0%)	25 (69.4%)	18 (48.6%)
Home blood sampling	18 (22.5%)	0 (0.0%)	1 (20.0%)	9 (25.0%)	8 (21.6%)
Telemedicine	2 (2.5%)	0 (0.0%)	0 (0.0%)	1 (2.8%)	1 (2.7%)
PPC outpatient clinic	10 (12.5%)	1 (50.0%)	1 (20.0%)	1 (2.8%)	7 (18.9%)
**Psychological counseling**	3 (3.8%)	0 (0.0%)	0 (0.0%)	0 (0.0%)	3 (8.1%)

Among non-oncologic patients, 821 activities were recorded, increasing from 52 in 2022 to 419 in 2025; outpatient clinic visits represented the largest share (*n* = 295; 36.0%), followed by home visits (*n* = 220; 26.8%), telemedicine (*n* = 161; 19.6%), psychological counseling (*n* = 104; 12.7%), and home blood sampling (*n* = 41; 5.0%). Among oncologic patients, 80 activities were recorded, increasing from 2 in 2022 to 37 in 2025; home visits were the most frequent activity (*n* = 47; 58.8%), followed by home blood sampling (*n* = 18; 22.5%) and outpatient visits (*n* = 10; 12.5%). At patient level, oncologic patients had more home visits (4.3 vs. 2.8) and home blood sampling procedures (1.6 vs. 0.5), whereas non-oncologic patients had more outpatient visits (3.7 vs. 0.9), psychological counseling (1.3 vs. 0.3), and telemedicine contacts (2.0 vs. 0.2) per patient. Private service use was recorded in 20 patients (22.0%), exclusively among non-oncologic patients, suggesting that some long-term supportive needs may not be fully covered by the public regional network.

### Observed versus expected regional need

The estimated total number of children requiring PPC in Friuli-Venezia Giulia ranged from 406 to 644, while 215 children were expected to require specialist PPC. The 91 observed specialist PPC patients corresponded to 42.3% of the expected regional specialist need. Coverage varied across LHAs, from 56.1% in ASUGI to 41.9% in ASUFC and 26.8% in ASFO (Table 3).

**Table 3:** Observed and expected pediatric palliative care need by LHA in Friuli Venezia Giulia (*ASFO*, Azienda Sanitaria Friuli Occidentale; *ASUFC*, Azienda Sanitaria Universitaria Friuli Centrale; *ASUGI*, Azienda Sanitaria Universitaria Giuliano Isontina; *FVG*, Friuli-Venezia Giulia)

		Children requiring PPC*				
		Expected		Observed	Coverage	
LHA	Population (31/12/2025)	PPC needs	PPC specialized	PPC specialized	PPC needs** (min–max)	PPC specialized
ASUGI	366.557	125–198	66	37	19%–30%	56%
ASFO	311.114	106–168	56	15	9%–14%	27%
ASUFC	515.825	175–279	93	39	14%–22%	42%
*FVG*	***1.193.496***	***406–644***	***215***	***91***	***14%–22%***	***42%***

*Abbreviations*: *PPC*, pediatric palliative care; *LHA*, Local Health Authority; *FVG*, Friuli-Venezia Giulia.

Annotations; *per 100,000 inhabitants; **calculated on total children requiring PPC.

### Care pathways

Two descriptive care pathways emerged according to subgroup conditions (Table 4). Non-oncologic patients represented a clinically heterogeneous population, most characterized by neurological disability associated with a known genetic disease (35/80; 43.8%), followed by neurological disability associated with acquired conditions (13/80; 16.3%) and neurological disability alone (12/80; 15.0%). Their care pathway was broad, technology-dependent, and multidisciplinary, with greater device dependency (> 3 devices: 32.5% non-oncologic vs. 9.1% oncologic) and more frequent outpatient visits, telemedicine contacts, and psychological support. Oncologic patients included hematologic malignancy (1; 9.1%), CNS tumors (6; 54.5%), and non-CNS solid tumors (4; 36.4%), and showed a more intensive pattern of care, with a higher median number of healthcare accesses (68 [IQR 49–147] vs. 60 [34–93]) and greater use of home visits and home blood sampling, consistent with more concentrated high-intensity care. All patients met ACCAPED level 3 criteria, confirming high clinical and care complexity in both groups. Median annualized healthcare utilization was 12 accesses per patient-year (IQR 9–22) among non-oncologic patients and 16 (IQR 3–17) among oncologic patients. These patterns were considered descriptive care pathways.

**Table 4:** Distribution of underlying conditions by subgroup. Non-oncologic conditions were grouped according to the presence of one or more of the following: neurological disability, acquired conditions, neuromuscular disease, and known genetic diagnoses. Oncologic conditions were classified as hematologic malignancies, CNS tumors, or non-CNS solid tumors

Subgroup conditions	
1. Non-oncologic, *n* (%)	80 (100)
Acquired condition	10 (12.5)
Known genetic diagnosis	1 (1.2)
Acquired condition + known genetic diagnosis	1 (1.2)
Neurological disability	12 (15.0)
Neurological disability + acquired condition + known genetic condition	1 (1.2)
Neurological disability + acquired condition	13 (16.3)
Neurological disability + known genetic disease	35 (43.8)
Neuromuscular disease + known genetic disease	7 (8.8)
2. Oncologic, *n* (%)	11 (100)
Hematologic malignancy	1 (9.1%)
CNS tumor	6 (54.5%)
Non-CNS solid tumor	4 (36.4%)

## Discussion

This regional real-world study provides a patient-centered reconstruction of specialist PPC pathways within an Italian Hub-and-Spoke network. To the best of our knowledge, this is among the first studies to reconstruct PPC delivery from the perspective of the patient journey across multiple care settings. By moving beyond single-setting descriptions, our findings highlight PPC as a longitudinal and multidisciplinary care pathway rather than a single specialist intervention.

Three main findings emerged. First, children receiving specialist PPC showed substantial clinical complexity, technological dependency, and intensive multidisciplinary healthcare utilization. Second, despite the Hub-and-Spoke organizational model, care delivery remained markedly centralized toward the regional tertiary Hub. Third, specialist PPC coverage remained incomplete and showed substantial variability across LHAs. Together, these findings highlight the close interaction between patient complexity, patterns of service utilization, and the organizational structure of the regional PPC network.

The study population reflects the growing relevance of CMC within pediatric healthcare systems. These children are medically fragile, frequently technology-dependent, and require multiple healthcare services over time. In the present cohort, nearly one third depended on more than three medical devices. This finding supports the need to integrate PPC planning with the broader management of CMC, for whom fragmented services, repeated transitions, and coordination failures may negatively affect continuity and equity of care. Within this population, oncologic and non-oncologic patients showed different patterns of care. This distinction was also supported by the underlying diagnostic profiles, with non-oncologic patients showing marked clinical heterogeneity dominated by neurological and genetic conditions, while the oncologic subgroup was mainly represented by CNS and non-CNS solid tumors. Non-oncologic patients represented most of the cohort and generated the largest volume of contacts. Their pathways were characterized by greater technological dependency and broad multidisciplinary involvement, including pediatric, emergency, surgical, neuropsychiatric, rehabilitation, outpatient, and telemedicine services. In contrast, oncologic patients showed higher healthcare utilization per patient and greater use of home visits and home blood sampling, with care more concentrated around hemato-oncology, treatment-related services, and high-intensity phases of care. These differences suggest that specialist PPC should not be organized as a uniform pathway, but should remain flexible enough to address both prolonged, technology-dependent care needs and more concentrated periods of high-intensity care. The comparison between observed and expected need is particularly relevant for health service planning. It is estimated that 34–54 children per 100,000 inhabitants in Italy require PPC and that approximately 18 per 100,000 require specialized PPC. Applying these benchmarks to Friuli-Venezia Giulia, the observed Hub cohort accounted for only 42.3% of the estimated specialist PPC need [19]. Coverage also varied substantially across LHAs, suggesting that under-identification, delayed referral, geographical proximity to the Hub, and differences in territorial capacity may influence access to specialist PPC. A regional PPC registry, shared eligibility criteria, and proactive case finding could support earlier identification, reduce territorial variability, and provide a stronger basis for resource allocation. The observed patterns of healthcare utilization should also be interpreted in relation to the organization of the regional network.

The presence of a single specialist Hub may favor concentration of multidisciplinary expertise, standardized assessment, complex care planning, training, and network coordination, but may also contribute to hospital-centered care and territorial mobility. Strengthening spoke capacity, shared protocols, telemedicine, home-based monitoring, and joint Hub-and-Spoke case management may allow a larger proportion of care to be delivered closer to the child’s place of residence while maintaining specialist oversight. Specialist and territorial nursing capacity may be particularly relevant in this context by supporting home-based monitoring, caregiver training, management of technological dependencies, and early recognition of clinical deterioration, potentially reducing avoidable hospital-based care.

These findings are also likely to vary according to the organizational model in which PPC is delivered. More centralized systems may generate greater tertiary-center utilization and travel burden for families, whereas integrated hospital–home models may result in a higher proportion of territorial contacts. Similarly, systems relying mainly on hospital consultation teams or general pediatric services without dedicated specialist PPC teams may show different patterns of care intensity, continuity, and resource distribution. Therefore, the specific volumes and distribution of healthcare utilization observed in Friuli-Venezia Giulia should not be directly generalized to other settings. However, the broader principles emerging from this study may be transferable across different organizational models. Equity also deserves specific attention. Foreign citizens represented more than one quarter of the cohort, a relevant finding in a population requiring repeated and complex interactions with the healthcare system. Caregivers with limited language proficiency may experience communication barriers associated with missed appointments, delayed services, medication-related problems, and difficulties navigating care pathways [22]. Such barriers may be particularly relevant in PPC, where communication, shared decision-making, advance care planning, and family support are central. Beyond language, religious beliefs and family traditions may also influence perceptions of illness, prognosis, and care preferences. Private service use, observed exclusively among non-oncologic patients, may further indicate that some long-term supportive, rehabilitative, or nursing needs are only partially covered by the public regional network. This is consistent with evidence that families of CMC may incur out-of-pocket expenses despite publicly funded healthcare systems [23].

This study has several limitations. Its retrospective design and reliance on records from a tertiary referral center mean that it captured patients formally taken in charge by the specialist PPC service but not necessarily all children with potential PPC needs in the region. Nursing activities were not consistently recorded as a separate category and could not be independently quantified, potentially underestimating their contribution to home care and hospital avoidance. Data completeness depended on routine clinical documentation, and activities delivered outside the public regional system may have been under-recorded. Moreover, the analysis focused on service volumes and pathway flows rather than patient-reported outcomes, family experience, quality of life, or appropriateness of individual healthcare contacts. Expected PPC need was estimated from published prevalence figures applied to regional population data and should therefore be interpreted as an approximate benchmark rather than a direct measure of unmet need. Finally, although the patient-centered pathway reconstruction approach may be transferable across healthcare systems, the observed resource-utilization patterns reflect the specific characteristics of this regional Hub-and-Spoke network and may differ in other organizational models.

## Conclusion

This study shows that specialist PPC in Friuli-Venezia Giulia involves mostly children with high clinical complexity. By reconstructing care from a patient-centered pathway perspective, it highlights two distinct trajectories: prolonged, technology-dependent, multidisciplinary pathways among non-oncologic patients and more time-concentrated, high-intensity pathways among oncologic patients. The marked centralization toward the tertiary Hub, the incomplete coverage of expected regional need, and the high proportion of foreign citizens supports the need to strengthen territorial PPC capacity, proactive case identification, culturally sensitive and spiritually aware family support, and integrated Hub-and-Spoke governance.

## Supplementary Information

Below is the link to the electronic supplementary material.

ESM 1 (DOCX 238 KB)

## Data Availability

No datasets were generated or analysed during the current study.

## Abbreviations

*ACCAPED*: Scheda di Accertamento dei Bisogni Clinico-Assistenziali Complessi in Pediatria
*ASFO*: Azienda Sanitaria Friuli Occidentale
*ASUFC*: Azienda Sanitaria Universitaria Friuli Centrale
*ASUGI*: Azienda Sanitaria Universitaria Giuliano Isontina
*ATS*: Association for Children with Life-Threatening or Terminal Conditions and their Families
*CMC*: Children with medical complexity
*CNS*: Central nervous system
*CPAP*: Continuous positive airway pressure
*CVC*: Central venous catheter
*ED*: Emergency department
*FVG*: Friuli Venezia Giulia
*HFNC*: High-flow nasal cannula
*IQR*: Interquartile range
*IRCCS*: Istituto di Ricovero e Cura a Carattere Scientifico
*ISTAT*: Istituto Nazionale di Statistica
*LHA*: Local Health Authority
*NIV*: Non-invasive ventilation
*PaPaS*: Paediatric Palliative Screening Scale
*PPC*: Pediatric palliative care
