# Supplementary material for: Pediatric palliative care in practice: real-world service utilization and care pathways in a regional Italian health system
Source: Eur J Pediatr. 2026 Sep 8;185(10):728. doi: 10.1007/s00431-026-07386-4 (PMC13553707; doi:10.1007/s00431-026-07386-4)
Supplement: Supplementary file 1 [file 431_2026_7386_MOESM1_ESM.docx]

**Supplementary materials**

**Figure S1:** Regional Hub-and-Spoke model for pediatric palliative care in Friuli Venezia Giulia

**Figure S2:** Flow chart of patient’s selection.

**Figure S3:** Interactive Sankey diagram available at the following link: <https://giuliazamagni.shinyapps.io/Sankey_services/>.

**Figure S4:** Mean number of PPC accesses per patient in oncologic versus non-oncologic patients, stratified by type of PPC activity.
